# Does endo-tracheal tube clamping prevent air leaks and maintain positive end-expiratory pressure during the switching of a ventilator in a patient in an intensive care unit? A bench study

**DOI:** 10.1371/journal.pone.0230147

**Published:** 2020-03-11

**Authors:** Emanuele Turbil, Nicolas Terzi, Carole Schwebel, Martin Cour, Laurent Argaud, Claude Guérin

**Affiliations:** 1 Anesthesiology and Intensive care, Università degli Studi di Sassari, Sassari, Italy; 2 Médecine Intensive Réanimation, C.H.U de Grenoble-Alpes, Grenoble, France; 3 University of Grenoble-Alpes, Grenoble, France; 4 Médecine Intensive-Réanimation, Hôpital Edouard Herriot, Hospices Civils de Lyon, Lyon, France; 5 University of Lyon, Lyon, France; 6 INSERM, Créteil, France; San Gerardo Hospital, ITALY

## Abstract

**Objectives:**

When patients with acute respiratory distress syndrome are moved out of an intensive care unit, the ventilator often requires changing. This procedure suppresses positive end expiratory pressure and promotes lung derecruitment. Clamping the endotracheal tube may prevent this from occurring. Whether or not such clamping maintains positive end-expiratory pressure has never been investigated. We designed a bench study to explore this further.

**How the study was done:**

We used the Elysee 350 ventilator in ‘volume controlled’ mode with a positive end-expiratory pressure of 15 cmH2O, connected to an endotracheal tube with an 8 mm internal diameter inserted into a lung model with 40 ml/cmH2O compliance and 10 cmH2O/L/s resistance. We measured airway pressure and flow between the distal end of the endotracheal tube and the lung model. We tested a plastic, a metal, and an Extra Corporeal Membrane Oxygenation clamp, each with an oral/nasal, a nasal, and a reinforced endotracheal tube. We performed an end-expiratory hold then clamped the endotracheal tube and disconnected the ventilator. We measured the change in airway pressure and volume for 30 s following the disconnection of the ventilator.

**Results:**

Airway pressure decreased thirty seconds after disconnection with all combinations of clamp and endotracheal tube. The largest fall in airway pressure (-17.486 cmH_2_O/s at 5 s and -18.834 cmH_2_O/s at 30 s) was observed with the plastic clamp combined with the reinforced endotracheal tube. The smallest decrease in airway pressure (0 cmH_2_O/s at 5 s and -0.163 cmH_2_O/s at 30 s) was observed using the Extra Corporeal Membrane Oxygenation clamp with the nasal endotracheal tube.

**Conclusions:**

Only the Extra Corporeal Membrane Oxygenation clamp was efficient. Even with an Extra Corporeal Membrane Oxygenation clamp, it is important to limit the duration the ventilator is disconnected to a few seconds (ideally 5 s).

## Introduction

Moving patients on invasive mechanical ventilation out of the intensive care unit (ICU) often requires switching them from a standard ICU ventilator to a portable one. In order to avoid lung derecruitment, clamping the endotracheal tube (ETT) represents a common practice in some institutions [1]. The same procedure is performed again when the patient is to be reconnected to the ICU ventilator. Worsening gas exchange or respiratory mechanics have been reported after the transportation of ICU patients [2–6]. Several factors can play a role in these changes. Changing the patient’s position from semi-recumbent to flat [7] in the Computer Tomography (CT) scanner can reduce end-expiratory lung volume [8]. The use of a portable ventilator, with a lower performance than the ICU ventilators in terms of tidal volume delivery and positive end expiratory pressure (PEEP), may result in hypoventilation and/or lung derecruitment [9–11]. Another factor is air leaks that may occur after the procedure described above, i.e. the clamping of the ETT followed by disconnection from the ventilator, should the clamping be only partly effective. This loss of lung volume could be particularly dangerous for patients with acute respiratory distress syndrome (ARDS) in whom loss of aeration is a key feature [12] and in whom PEEP [13, 14] is recommended. Moreover, some authors have reported clamping the ETT during experimental procedures. For example, Lu et al compared the derecruited lung volume after PEEP removal, measured with a Pressure-Volume (VP) curve, with that measured with CT clamping of the ETT during image acquisition [15].

The role of leaks after clamping the endotracheal tube and ventilator disconnection has not previously been studied. Therefore, we designed a bench study to investigate whether clamping the ETT during an expiratory hold would efficiently prevent volume loss. The working hypothesis was that the plastic clamp would be, and the metal clamp might be, inefficient in maintaining PEEP and end-expiratory lung volume. Our secondary aim was to investigate whether the clamping maneuver would increase ETT resistance.

## Middle section

### Material and methods

We used the Elysee 350 ICU ventilator (ResMed, San Diego, California) connected to an 8 mm ID ETT inserted into a lung model (ASL 5000, Ingmar inc., Pittsburgh). The lung model was set in passive condition with a compliance of 40 ml/cmH_2_O and a resistance of 10 cmH_2_O/L/s. Two flow meters (3700 series, Hans-Rudolph, Shawnee, Kansas) and two airway pressure (Paw) ports were placed at each tip of the ETT.

The pressure ports were connected to a piezoresistive transducer (BD Gabarith, Vogt Medical, Vertrieb, Karlsruhe, Germany). The pneumotachographs were calibrated using a constant flow and a precision rotameter (Houdec Glass, Martin Medical, Lyon, France), while the pressure transducers were calibrated against a manometer (717 1G, Fluke Biomedical, Everett, Washington). A data logger (MP150, Biopac Systems, Goletta, California) was used for recording flow and airway pressure.

Three different clamps were used: a plastic clamp (Kangao inc., Jiangsu, China), a metal clamp (Prestige Medical inc., Raincy, France) and an ECMO clamp (Landanger inc., Paris, France).

Three kinds of brand new ETTs, all used in the Grenoble-Alpes University hospital, were investigated: oral/nasal tube (Shiley Hi-Contour Oral/Nasal Tracheal Tube Cuffed, Covidien, Mansfield, MA), nasal tube (Mallinckrodt Nasal RAE tracheal tube cuffed, Covidien Mansfield, MA) and reinforced tube (ShileyLo-contour Oral/nasal tracheal tube cuffed, Covidien, Mansfield, MA).

### Protocol

The experiment consisted of four steps for each clamp and each kind of ETT (Fig 2). In each condition the ETT was put into a connector and then the cuff was gently inflated to stabilize the ETT. We gently manually tested the tight position of the ETT onto the connector.

**Fig 1:**
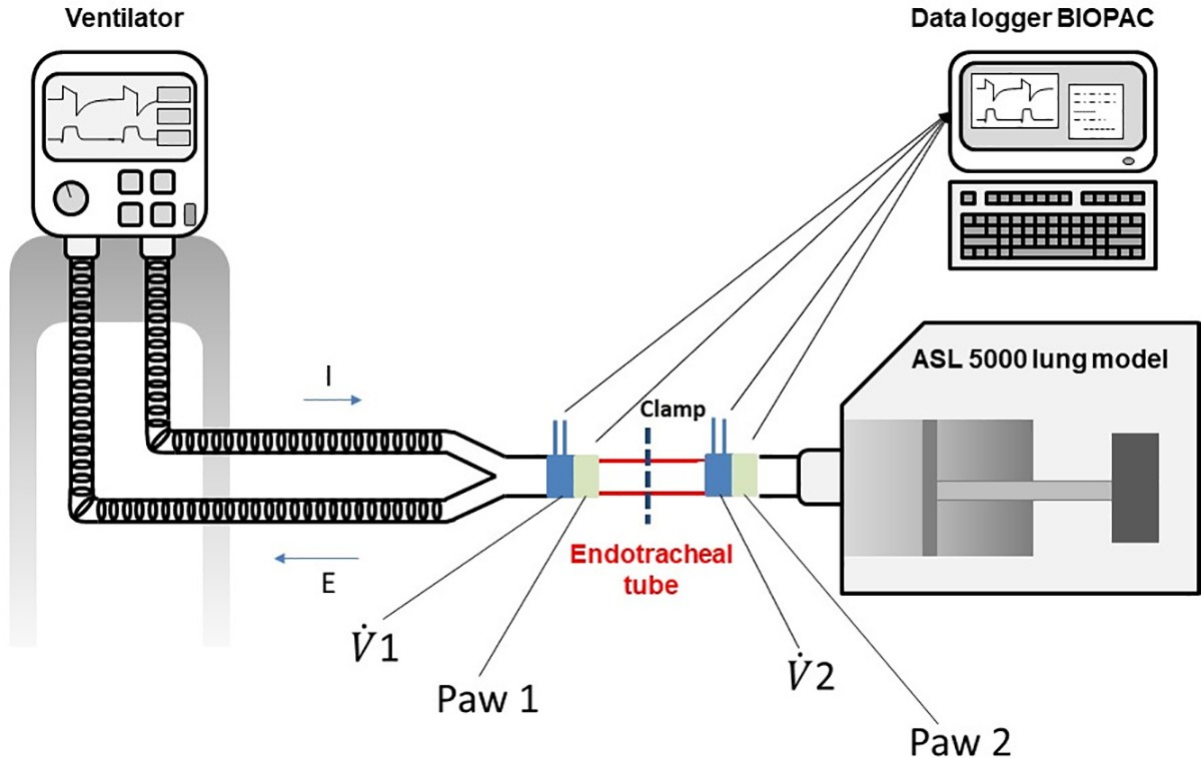
Experimental set up. $\dot{V}1$ **= flow measured with pneumotachograph number 1,** $\dot{V}2$ **= flow measured with pneumotachograph number 2, Paw 1 = pressure measured with pressure transducer number 1, Paw 2 = pressure measured with pressure transducer number 2.** Arrows indicate the connection between Paw ports and flow meters to data logger. The location of the clamp into the endotracheal tube is shown. I = inspiration, E = expiration.

**Fig 2:**
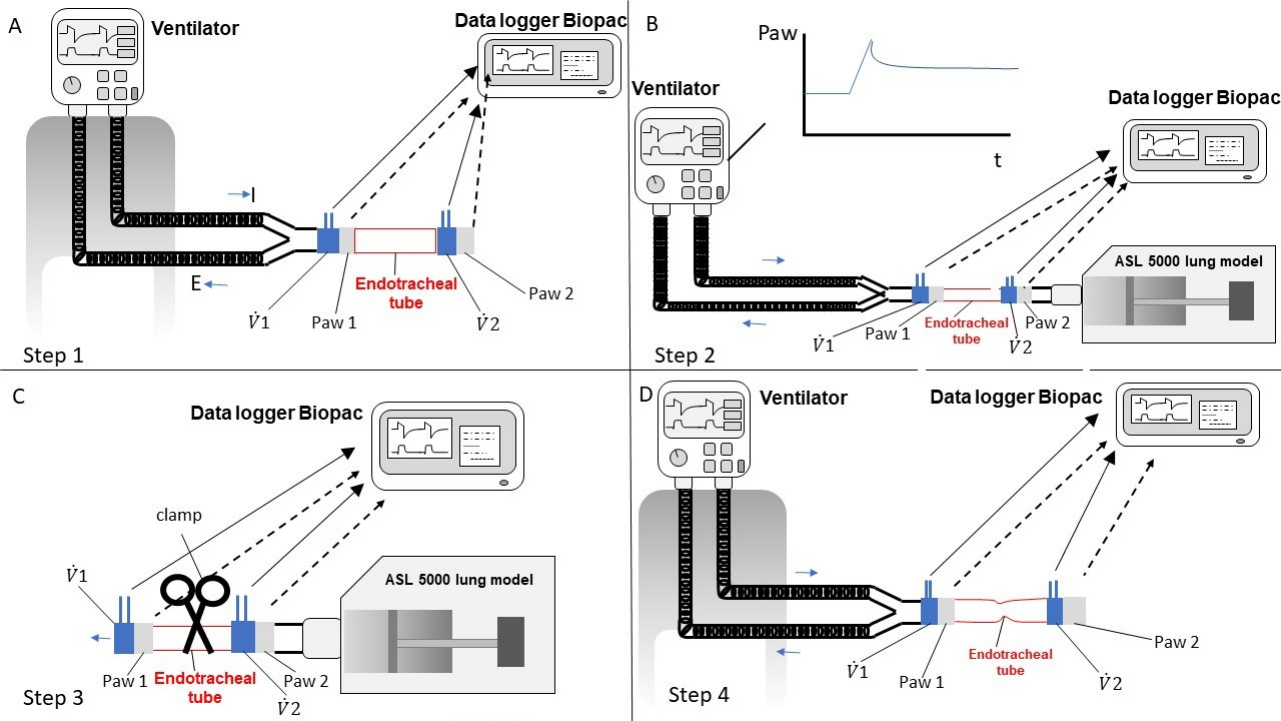
The four steps of the experiment are shown. A) Step 1, measurement of the endotracheal tube resistance before clamping; B) Step 2, inspiratory hold to detect air leaks in the entire set up; C) Step 3, endotracheal tube clamping, disconnection from the ventilator and measurement of airway pressure and flow over time; D) Step 4, measurement of the endotracheal tube resistance after clamping; Paw = airway pressure; t = time; $\dot{V}1$ = flow measured with pneumotachograph number 1 (as in Fig 1); $\dot{V}2$ = flow measured with pneumotachograph number 2 (as in Fig 1); Paw 1 = pressure measured with pressure transducer number 1 (as in Fig 1); Paw 2 = pressure measured with pressure transducer number 2 (as in Fig 1). Horizontal blue thin arrows indicate the inspiratory (I) and expiratory (E) direction. Black arrowed lines show the connection between the data logger and the Paw ports (broken lines) and flow meters (continuous lines).

First, we measured the ETT airflow resistance prior to any manipulation by connecting the ventilator to the set-up shown in Fig 1 and removing the ASL 5000 and leaving the distal tip in the air (Fig 2A). We subsequently varied the squared insufflation flow from the ventilator between 0.2 L/s and 1.2 L/s and recorded flow and Paw (Fig 2A).

In the second step, we assessed the presence of air leakages within the whole set-up. To do this, we connected the ETT to the whole set-up (Fig 2B). We then placed the ventilator in volume controlled mode with a constant flow and with a tidal volume of 400 ml, a respiratory rate of 21 breaths/min, a PEEP of 15 cmH_2_O, an FIO_2_ of 21% and an Inspiratory/Expiratory time ratio of 1:2. We then delivered 4 breaths before performing a 15 s inspiratory hold to assess the presence of a leak in the set-up. We excluded the presence of air leakage if, during the pause, the Paw plateaued over time.

For the third step we performed an expiratory hold, then clamped the ETT and detached the ventilator from the ETT for at least 30 s. ETT was clamped 5 cm from the connector. The clamp was applied at a right angle to the ETT. During this time, we recorded the Paw and flow using the pneumotachograph and the Paw port located between the ETT and the lung model (i.e. Paw2 and flow 2 In Fig 2C).

Finally, the fourth step consisted of repeating the measurement of the ETT resistance after clamping (Fig 2D).

### Data analysis

**Fig 3:**
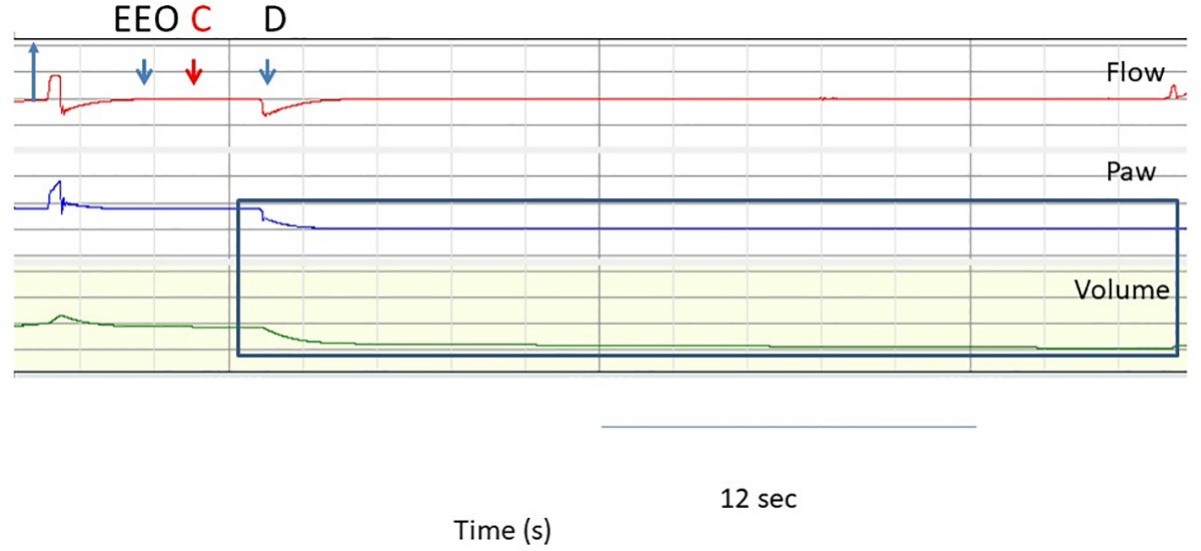
Criteria for defining and quantifying leaks after clamping the endotracheal tube (ETT) and disconnecting the ventilator. From top to bottom, records of flow, airway pressure (Paw) and volume during the clamping of the nasal tube with the plastic clamp over time. Flow, Paw and volume were recorded between the lung model and the ETT. The first blue arrow indicates the inspiratory direction. The second blue arrow (EEO) shows the expiratory hold, the red arrow indicates the time of ETT clamping and the third blue arrow shows the disconnection of the ventilator from the ETT and the lung model. Paw and volume tracings after the clamping are shown in the blue rectangle. It can be seen that the Paw dropped after the detachment of the ventilator. The ventilator was then reconnected and baseline ventilation resumed. EEO = end expiratory occlusion. C = ETT clamping. D = disconnection of the ventilator from ETT.

**Fig 4:**
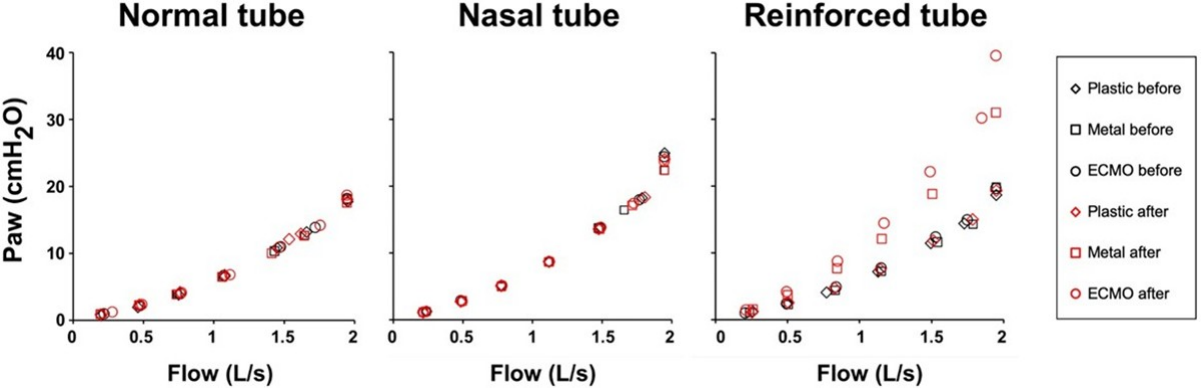
Relationship between airway pressure and flow before and after clamping of a nasal, oral and reinforced tube with each clamp. ECMO = extracorporeal membrane oxygenation.

The primary endpoint was the Paw decay 5 s and 30 s after disconnection, while the secondary endpoints were the volume lost (Vlost) and the change in ETT resistance after clamping over the same period. Paw decay was measured as the Paw change at 5 s and 30 s from the time just before disconnection divided by the time after disconnection and expressed as cmH2O/s (Fig 3). To assess the Vlost we integrated the flow curve over 5 s and 30 s (Fig 4). Finally, ETT resistance was obtained by fitting the equation Paw = k1 flow + k2 flow^2^ to the Paw-flow relationship. Paw at 1 L/s was equal to k1+k2 and taken as the ETT resistance at a flow of 1 L/s (Fig 4).

Data were analyzed with R 3.2.0 software (R Foundation for Statistical Computing, Vienna, Austria), using ANOVA with pairwise comparisons with the Holm procedure. Results are shown as medians (1^st^-3^rd^ quartiles). P values <0.05 were considered statistically significant.

## Results

### Paw decay and volume loss

**Fig 5:**
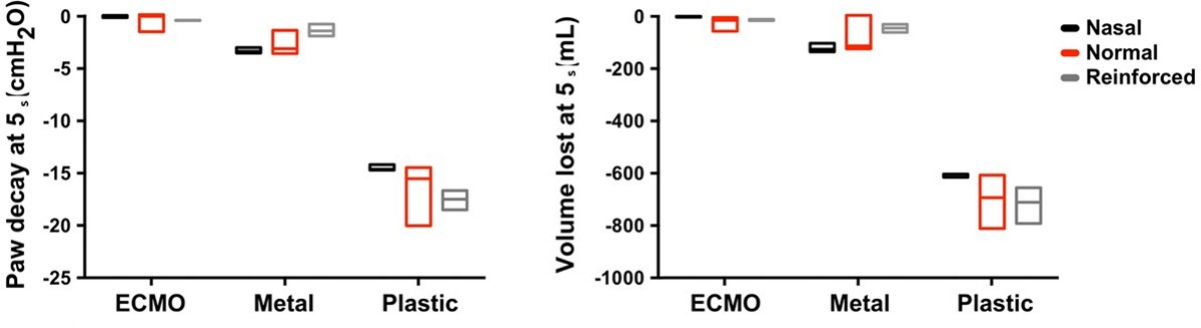
Airway pressure (Paw) decay and volume lost after 5 s following endotracheal tube clamping and set up disconnection.

**Fig 6:**
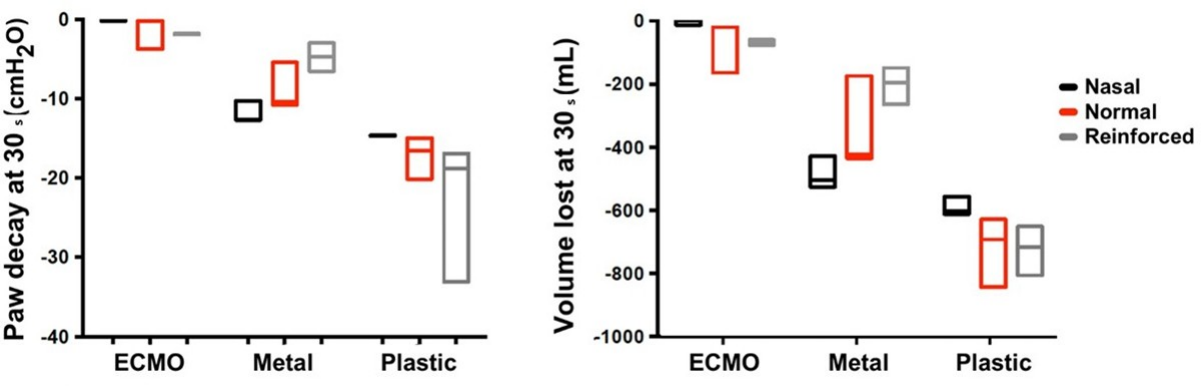
Airway pressure (Paw) decay and volume lost after 30 s following endotracheal tube clamping and set up disconnection.

Both the ETT and the clamp had a significant effect on Paw decay and Vlost (Table 1) but their interaction was also significant. At 5 s, all the combinations of nasal and oral ETTs and clamps, except for the ECMO clamp, showed a Paw decay (Table 1 and Figs 5 and 6). At 30 s post disconnection, Paw decayed with all combinations of clamps and ETTs, except for the ECMO clamp with the Nasal and Oral ETTs (Table 1 and Fig 6). The largest Paw decay (-17.486 cmH2O/s at 5 s and -18.834 cmH_2_O/s at 30 s) was observed with the plastic clamp combined with the reinforced ETT. The smallest Paw decay (0 cmH_2_O/s at 5 s and -0.163 cmH_2_O/s at 30 s) was observed using the ECMO clamp with the nasal ETT. Total Paw loss was only observed with the plastic clamp. For the nasal, oral and reinforced tubes, the time from disconnection to total loss of Paw was 2.3 s (2.25 s-2.42 s), 3.00 s (2.95 s-3.03 s) and 2.18 s (2.08 s-2.45 s), respectively.

**Table 1:** Airway pressure decay and volume lost 5 seconds and 30 seconds after disconnecting the endotracheal tube clamped for the three kinds of endotracheal tube.

ETT	Clamp	Paw decay (cmH2O/s) 5 sec*,**	Volume lost (L) 5 sec*,**	Paw decay (cmH2O/s) 30 sec *,**	Volume lost (L) 30 sec *,**
Nasal	ECMO	0.000 (-0.061;0.040)	0.000 (0.000; -0.002)	-0.163 (-0.183;-0.082)	0.000 (0.000;-0.007)
	Metal	-3.350 (-3.431;-3.166) †	-0.126 (-0.115; -0.131) †	-12.624 (-12.665;-11.460) †	-0.504 (-0.466;-0.515) †
	Plastic	-14.626 (-14.666;-14.401) †‡	-0.603 (-0.603; -0.609) †‡	-14.707 (-14.707;-14.667) †	-0.602 (-0.579;-0.607) † ‡
Oral	ECMO	0.000 (-0.735; 0.081)	-0.015 (-0.001; 0.036)	-0.204(-1.981;-0.204)	-0.020(-0.020;-0.092)
	Metal	-3.105 (-3.330;-2.227)	-0.113 (-0.055;-0.119)	-10.377 (-10.581;-7.905)	-0.423 (-0.298;-0.429) †
	Plastic	-15.525 (-17.772;-14.994) †‡	-0.693 (-0.650;-0.752) †‡	-16.587 (-18.364;-15.770) †	-0.692 (-0.660;-0.768) † ‡
Reinforced	ECMO	-0.367 (-0.388;-0.367)	-0.015(-0.013;-0.016)	-1.797(-1.838;-1.797)	-0.076(-0.068;-0.077)
	Metal	-1.389 (-1.634;-1.062)	0.045 (-0.037;-0.053)	-4.698 (-5.617;-3.840)	-0.195 (-0.172;-0.229) †
	Plastic	-17.486 (-17.996;-17.078) †‡	-0.711 (-0.683;-0.752) †‡	-18.834 (-25.963;-17.874) †	-0.716 (-0.684;-0.761) †‡

Paw = airway pressure, ETT = endotracheal tube, ECMO = extracorporeal membrane oxygenation

Values are median (1^st^-3^rd^ quartiles)

*P <0.001 for clamp effect

** P <0.001 for the interaction between the ETT and clamp

†P <0.001 vs. ECMO

‡P <0.001 vs. Metal

Vlost was consistent with Paw decay findings (Table 1 and Figs 5 and 6). The worst performance was with the plastic clamp combined with the reinforced ETT. In this case, a Vlost of -0.826 L at 5 s and -1.011 L at 30 s were observed. The smallest Vlost was associated with the ECMO clamp combined with the nasal ETT, which resulted in a value of -0.027 L at 5 s and -0.157 L at 30 s.

### ETT resistance

The Paw-flow relationships were not different across tubes before clamping (Fig 4). The ETT resistance at 1 L/s was greater after clamping than before for both the ECMO and the metal clamp when applied to the reinforced ETT (Table 2). A statistical comparison was not performed because we only measured ETT resistance once. However, the external diameter of the reinforced ETT was permanently narrowed by the clamping.

**Table 2:** Airflow resistance of the endotracheal tube before and after clamping for different tubes and clamps.

Endotracheal tube	Clamp	Time of clamping	Endotracheal tube airflow resistance (cmH2O/L/s)	Change in Endotracheal tube airflow resistance: [after—before clamping] (cmH2O/L/s)
Nasal	ECMO	Before	7.2	-0.1
		After	7.1	
Normal	ECMO	Before	5.9	-0.1
		After	5.8	
Reinforced	ECMO	Before	6.2	4.8
		After	11	
Nasal	Plastic	Before	7.2	0.1
		After	7.3	
Normal	Plastic	Before	5.9	0.3
		After	6.2	
Reinforced	Plastic	Before	6.0	0.1
		After	6.1	
Nasal	metal	Before	7.3	-0.1
		After	7.2	
Normal	metal	Before	5.9	-0.1
		After	5.8	
Reinforced	metal	Before	5.6	3.9
		After	9.5	

ECMO: extracorporeal membrane oxygenation

## Discussion

The main finding of our study is that the type of ETT and the type of clamp both have a significant impact on PEEP stability and loss of lung volume after ETT clamping and ventilator disconnection.

With regard to the prevention of leaks, the ECMO and the plastic clamp had the best and worst performance, respectively, while the metal clamp was in between. These results are probably due to the size of the ECMO clamp, which allows a stronger clamping. As shown in Fig 2, the metal clamp is only 14 cm long while the ECMO clamp is about 21 cm long, which provides a greater lever effect and hence a better grip on the ETT. The plastic clamp resulted in an extremely large air leakage. Furthermore, with a Paw decay of about 18 cmH2O/s, it would only take a few seconds to lose all the PEEP and end expiratory lung volume. Based on the present findings, the use of the plastic and metal clamp should be contraindicated.

It should be mentioned that the maximum drop in pressure due to inadequate clamping was higher than the set PEEP value because, firstly, the measured PEEP value may be slightly different from the PEEP value set on the ventilator, and secondly, and more importantly, auto-PEEP was generated by the endotracheal tube after clamping.

We also measured the impact of clamping on the ETT resistance. The ETT resistance only increased after the clamping of the reinforced ETT. This is probably due to the shape memory of this kind of ETT. This phenomenon has previously been described in some case reports where, after a patient had bitten the ETT, an increased peak pressure during ventilation and a significant desaturation were observed [16, 17]. Care should be taken when using this type of ETT for long-term use during mechanical ventilation in the ICU, except for specific purposes, due to the increased ETT resistance after clamping. The reinforced ETT is currently indicated for surgeries where the anesthesiologist cannot access the patient’s airways, e.g. craniectomy, neck and head surgery or spine surgery [17–19]. Sometimes, the patient needs to be admitted to the ICU after this kind of surgery and the mechanical ventilation has to continue. In this situation, it is important to know that a reinforced tube has a well-known risk of permanent occlusion following a patient bite or, as demonstrated in our experiment, after ETT clamping. Therefore, in this scenario, it is important to consider the replacement of the reinforced ETT with a conventional ETT. Another indication for the use of a reinforced tube is intubation through a fast track laryngeal mask in the event of an unanticipated difficult intubation [20, 21]. In this case, the potential risks of maintaining a reinforced ETT are probably lower than the ones linked to changing the ETT in a patient with difficult airways. Therefore, reinforced tube should not be clamped.

### Clinical implications

PEEP is a key setting in ARDS patients and its maintenance over time should be an important target for clinicians. Some studies have shown that the ability of the ICU ventilators to deliver the set PEEP varies [9–11]. Furthermore, it has been shown in both a lung model [22] and in real patients [23] that a routine maneuver, such as tracheal suctioning, can cause PEEP to drop. Therefore, it is strongly recommended that lung derecruitment be prevented during tracheal suctioning to avoid abrupt disconnection from the ventilator or to use a specific device that maintains some lung volume throughout the procedure. In this paper we evaluated another condition with the potential of lung derecruitment and proved the presence of air leaks resulting from the clamping of the ETT.

### Limitations and strengths

The major strength of this study is the fact that we used an experimental model with standardized procedures to test our hypothesis. Mechanical ventilation was constant for the entire length of the experiment, while the type of ETT and clamp used were the only variable factors. We also used a value of compliance that is realistic for an ARDS patient. Another strength is the measurement of Paw and flow rate between the lung model and the ETT, which is very difficult or almost impossible to assess in a real patient. We focused our design on a passively mechanically ventilated patient and did not consider the patient breathing spontaneously. Clamping the ETT in a patient with inspiratory efforts, even to avoid lung derecruitment, is not recommended because the effort of breathing will increase dramatically and lead to dyspnea [24].

However, even though the ASL lung model is very realistic, it is not a real lung. We mentioned above that our clamping may mimic a patient’s bite. This latter would occur in patients with spontaneous inspiratory effort. As previously indicated, we did not explore this in our study. Another possible difference from a real patient is that the leakage can also occur in the ETT cuff. In our experiment, the cuff was placed and inflated in such a way that this type of leak was avoided. In a real patient, the cuff is inflated to about 25 cmH_2_O [25–27]. In this situation, the manipulation of the ETT during clamping would increase the air leakage around the cuff. Even so, it is hard to think that the use of different types of clamps can influence the amount of leakage around the cuff. Finally, our experiment could not assess whether handling during ETT clamping and ventilator disconnection would favor unscheduled extubation. Cephalad migration of the ETT has been reported during ETT handling in a clinical setting [28, 29]. As ETTs are thermable, it is highly likely that the results in patients would be different when the tubes are warmed by body temperature.

We only studied 3 clamps and 3 ETTs, which limits the generalizability of our data as a huge variety of clamps and ETTs can be found throughout the world.

## Conclusion

These results provided evidence that only the ECMO clamp was able to perform an efficient ETT clamping. In addition, we demonstrated that, even when an ECMO clamp is used, it is important to limit the duration of disconnection from the ventilator to a few seconds (ideally 5 s).

## Supporting information

S1 Data (PDF)

S2 Data (XLSX)
