# Supplementary material for: Does endo-tracheal tube clamping prevent air leaks and maintain positive end-expiratory pressure during the switching of a ventilator in a patient in an intensive care unit? A bench study
Source: PLoS One. 2020 Mar 11;15(3):e0230147. doi: 10.1371/journal.pone.0230147 (PMC7065807; doi:10.1371/journal.pone.0230147)
Supplement: S1 Data — (PDF) [file pone.0230147.s001.pdf]

ETT	Clamp	mesure	Pplat	Pawdecay	deltaT	slopePaw	PEEPtot	Paw5s
Oral	Plastic	1	25	-15.57	43.06	-0.361588481	15.075	0.49
Oral	Plastic	2	25	-14.626	44.992	-0.325080014	15.606	0.449
Oral	Plastic	3	26	-20.346	36.072	-0.564038589	20.754	0.449
Oral	Metal	1	24	-5.025	33.676	-0.149216059	14.013	12.256
Oral	Metal	2	25	-9.601	29.284	-0.327858216	15.075	12.665
Oral	Metal	3	26	-11.316	39.4	-0.287208122	15.03	11.643
Oral	ECMO	1	25	-0.326	38.272	-0.008517977	15.075	15.075
Oral	ECMO	2	25	-4.044	45.06	-0.089747004	15.075	13.972
Oral	ECMO	3	25	-0.326	44.328	-0.007354268	15.116	15.034
Nasal	Plastic	1	25	-14.707	29.44	-0.499558424	15.034	0.245
Nasal	Plastic	2	25	-14.626	29.376	-0.497889434	14.789	0.122
Nasal	Plastic	3	25	-14.381	20.964	-0.685985499	15.157	0.163
Nasal	Metal	1	29	-13.972	38.404	-0.363816269	18.548	15.034
Nasal	Metal	2	28	-13.278	36.208	-0.366714538	16.955	14.135
Nasal	Metal	3	28	-11.48	36.072	-0.318252384	17.159	14.789
Nasal	ECMO	1	30	-0.285	54.44	-0.005235121	19.079	19.038
Nasal	ECMO	2	29	-0.204	36.54	-0.005582923	18.017	17.935
Nasal	ECMO	3	29	-0.245	41.932	-0.005842793	18.384	18.344
reinforced	Plastic	1	36	-18.793	74.34	-0.252797955	18.75	-0.04
reinforced	Plastic	2	29	-17.567	59.836	-0.293585801	17.731	0.245
reinforced	Plastic	3	29	-17.077	60.232	-0.283520388	17.118	0.245
reinforced	Metal	1	30	-10.254	62.496	-0.164074501	19.448	18.058
reinforced	Metal	2	30	-7.231	61.764	-0.117074671	18.834	17.649
reinforced	Metal	3	28	-5.106	61.964	-0.082402685	17.486	16.873
reinforced	ECMO	1	24	-3.391	60.496	-0.056053293	15.034	14.544
reinforced	ECMO	2	24	-3.186	60.564	-0.052605508	14.707	14.299
reinforced	ECMO	3	25	-3.431	60.1	-0.057088186	14.626	14.054

Leak Paw crierion5s	Pawdecay5s	deltaT5sec	slopePaw5sec	Paw30s	Pawdecay30s	deltaT30s
1	-15.525	5.056	-3.070609177	0.531	-16.587	30.084
1	-14.462	5.06	-2.858102767	0.531	-14.953	30.416
1	-20.019	5.06	-3.956324111	0.49	-20.141	30.084
1	-1.348	5.06	-0.266403162	9.11	-5.433	30.684
1	-3.554	5.06	-0.702371542	5.27	-10.785	29.816
1	-3.105	5.056	-0.614121835	4.412	-10.377	30.016
0	0.163	5.056	0.032238924	14.83	-0.204	30.016
1	-1.47	5.056	-0.290743671	11.643	-3.758	30.016
0	0	5.06	0	14.953	-0.204	30.02
1	-14.707	5.06	-2.906521739	0.204	-14.707	29.44
1	-14.626	5.056	-2.892800633	0.081	-14.626	29.376
1	-14.176	5.06	-2.801581028	0.245	-14.707	17.304
1	-3.513	5.06	-0.694268775	5.842	-12.706	30.616
1	-3.35	5.056	-0.662579114	5.352	-12.624	30.02
1	-2.982	5.06	-0.589328063	7.476	-10.295	30.084
1	-0.122	5.056	-0.024129747	18.834	-0.204	30.084
0	0	5.06	0	17.812	0	30.084
0	0.081	5.06	0.016007905	18.18	-0.163	30.084
1	-18.507	5.06	-3.657509881	4.00E-02	-18.834	30.816
1	-17.486	5.056	-3.45846519	0.204	-33.092	30.02
1	-16.669	5.056	-3.296875	0.204	-16.914	30.016
1	-1.879	5.06	-0.371343874	13.4	-6.536	30.084
1	-1.389	5.06	-0.274505929	14.381	-4.698	30.02
1	-0.735	5.06	-0.145256917	14.667	-2.982	30.016
1	-0.367	5.056	-0.072587025	13.114	-1.797	30.08
1	-0.367	5.056	-0.072587025	12.828	-1.879	30.084
1	-0.408	5.06	-0.080632411	12.706	-1.797	30.084

slopePaw30s	Vlost5s_drift_corrected	Vlost30s_drift_corrected	DeltaPplatLeakTest	DeltaVTLeakTest
-0.551356203	0.693	0.692	-0.37	0
-0.491616255	0.607	0.628	0.081	0
-0.669492089	0.811	0.843	-0.2	0
-0.177062964	-0.004	0.174	-0.7	0
-0.36171854	0.113	0.423	-0.29	0
-0.345715618	0.124	0.435	-0.12	0
-0.006796375	0.004	0.02	-0.2	0
-0.125199893	0.057	0.164	-0.16	0
-0.00679547	0.015	0.02	-0.08	0
-0.499558424	0.615	0.612	-0.2	0
-0.497889434	0.603	0.602	-0.08	0
-0.849919094	0.603	0.556	-0.2	0
-0.415011759	0.135	0.526	-0.12	0
-0.420519654	0.126	0.504	-0.25	0
-0.342208483	0.103	0.428	-0.25	0
-0.006781013	0	0	-0.24	0
0	0.003	0.013	-0.08	0
-0.005418162	0	0	-0.44	0
-0.611176012	0.792	0.806	-0.5	0
-1.102331779	0.711	0.716	-0.3	0
-0.563499467	0.655	0.651	-0.2	0
-0.217258343	0.061	0.264	-0.5	0
-0.15649567	0.045	0.195	-0.5	0
-0.099347015	0.03	0.149	-0.5	0
-0.059740691	0.016	0.076	-0.8	0
-0.06245845	0.011	0.06	-0.8	0
-0.059732748	0.015	0.077	-0.9	0

